# Flap revascularization in patients following immediate reconstruction using an autologous free dermal fat graft for breast cancer: a report of two cases

**DOI:** 10.1186/s40792-016-0181-2

**Published:** 2016-06-03

**Authors:** Hiroaki Shima, Goro Kutomi, Takuro Kyuno, Fukino Satomi, Satoko Uno, Hideki Maeda, Hidekazu Kameshima, Tosei Omura, Yasutoshi Kimura, Toru Mizuguchi, Koichi Hirata, Ichiro Takemasa

**Affiliations:** Department of Surgery, Surgical Oncology and Science, Sapporo Medical University, S 1, W 16, Chuo-ku, Sapporo, 060-8543 Japan; Breast Cancer Center, Higashi-Sapporo Hospital, Higashi-Sapporo 3 Jou 3 Tyoume, Shiroishi-ku, Sapporo 003-8585 Japan; Department of Surgery, JR Sapporo Hospital, N3, E1, Chuo-ku, Sapporo, 060-0033 Japan

**Keywords:** Breast-conserving surgery, Tissue transplant, Breast reconstruction, Contrast-enhanced ultrasound

## Abstract

It has been reported that use of the free dermal fat graft (FDFG) technique produces a good cosmetic outcome for breast cancer. An FDFG is harvested from the lower abdomen as a columnar-shaped specimen and implanted into the defect of the breast after a partial mastectomy as a volume replacement technique. In this report, two patients who underwent breast-conserving surgery with immediate reconstruction using an autologous FDFG are described in order to show the difference in status between one case with and one without blood flow in the graft. To assess the benefit of this technique using FDFGs, their cosmetic satisfaction was evaluated using a questionnaire, graft shrinkage was measured by CT, and blood flow was assessed using contrast-enhanced ultrasound (CEUS). Both patients scored 10 of 12 points on the questionnaire. After 2 years, shrinkage of the grafts was 21.6 and 25.2 %, respectively. Although one patient had no blood flow in the center of the graft, the other had blood flow from the pectoralis major muscle to the center of the graft. While satisfaction and graft shrinkage were similar in the two patients, one case showed blood flow and had a somewhat softer graft than the other. The graft status was maintained with a good cosmetic outcome for 3 years after breast-conserving surgery with immediate reconstruction using an autologous FDFG, despite mild shrinkage and hardness of the graft. It is notable that blood flow was observed into the graft on CEUS, and more distinct perfusion was seen in the softer graft case after more than 3 years.

## Background

Currently, breast-conserving surgery (BCS) is a standard technique for early-stage breast cancer. This is because the long-term survival rate among women who undergo BCS is equivalent to the survival outcome in patients who undergo mastectomy [1, 2]. Immediate breast reconstruction is indispensable for the treatment of breast cancer [3]. For breast cancer patients, it is important to perform reconstruction using the optimal available procedures with respect to breast shape, breast size, and surgical defect location and size [3]. On the other hand, there are certain limitations to reconstruction using only a rotation and fixation technique of the parenchymal adipose tissue or gland when dealing with the varieties of breast shapes [4]. It has been reported that it might be more difficult to maintain symmetry after a partial mastectomy in Asian women, including Japanese women, than in Western women [5]. Deformed breasts may predominate, especially when a woman with small breasts undergoes repair by simple displacement after BCS [4–6]. Kijima et al. reported that immediate breast reconstruction using an autologous free dermal fat graft (FDFG) is one of the best reconstruction methods [7]. It has also been reported that use of the FDFG technique produces good cosmetic outcomes by adjoining the pectoralis major muscle, since revascularization can occur between these two surfaces [4, 5, 7, 8]. To the best of our knowledge, there have been no reports of the assessment of blood flow using contrast-enhanced ultrasound (CEUS). It has been reported that micro-bubbles used as ultrasound (US) contrast agents are retained only in the blood vessels. Recently, Sonazoid (perflubutane, Daiichi-Sankyo Pharmaceuticals, Nihonbashi-honcho, Chuo-ku, Japan) has been used as a second-generation US contrast agent. Sonazoid has been approved for use in diagnostic US for characterization and detection of focal breast lesions, and the micro-bubbles distribute in the micro-circulation of the whole body [9]. In this report, CEUS was added for the assessment of FDFG blood flow 3 years after surgery. Two early-stage breast cancer patients in whom BCS was performed using an FDFG with immediate reconstruction are described, and the blood flow of the graft and cosmetic appearance were evaluated.

The detail of the methods of assessing cosmetic satisfaction using a questionnaire is described below. Cosmetic statements made by the patients were evaluated objectively and subjectively using the method reported by Sawai’s group and supported by the Japanese Breast Cancer Society (JBCS) [10]. This assessment contains eight items: (1) breast size; (2) breast shape; (3) wound scar; (4) softness of the breast; (5) shape and size of the nipple-areola; (6) color of the nipple-areola; (7) level of the nipple (difference in the distance from the suprasternal notch for bilateral nipples); and (8) the lowest point of the breast (difference of bilateral breasts). Cosmetic outcomes were evaluated as excellent when the total score was 12 points, good when 9–11, fair when 5–8, and poor when 0–4. This assessment was completed by each patient herself by responding to the survey 2 years after surgery (Table 1). Any comments were added to the record.

**Table 1:** Assessment of the cosmetic results respond by patients themselves after surgery objective and subjective assessment (Japanese Breast Cancer Society)

		Case 1			Case 2		
	Total	6 months	1 year	3 years	6 months	1 year	3 years
Breast size	/2	2	2	2	2	2	2
Breast shape	/2	2	2	2	2	1	1
Wound scar	/2	1	1	1	1	1	2
Softness of the breast	/2	1	0	1	1	1	1
Shape and size of nipple-areola	/1	1	1	1	1	1	1
Color of nipple-areola	/1	1	1	1	1	1	1
Level of nipple (difference of distance from the suprasternal notch for bilateral nipples)	/1	1	1	1	1	1	1
The lowest point of the breast (difference of bilateral breasts)	/1	1	1	1	1	1	1
	/12	10	9	10	10	9	10

Cosmetic statements made by patients were evaluated objectively and subjectively, 6 months, 1 year, and 3 years after surgery. The cosmetic outcome was evaluated as excellent when the total score was 12 points, good when 9–11, fair when 5–8, and poor when 0–4 [9]. “Good” scores were obtained in both cases 2 years after surgery. “Softness of the breast” caused a decrease in points in both patients

## Case presentation

### Case 1

**Fig. 1:**
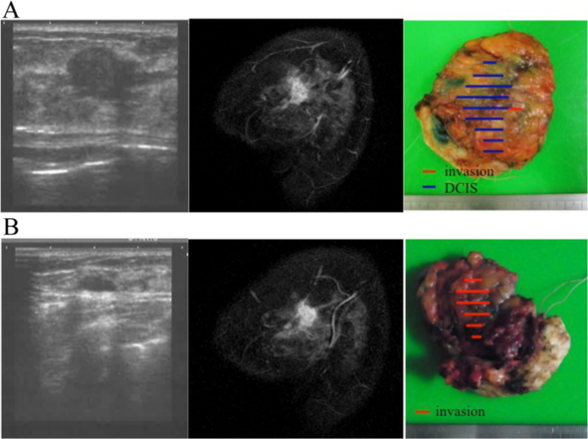
Ultrasound (US: *left panel*), magnetic resonance imaging (MRI: *center panel*), and pathological findings (*right panel*) of case 1 (**a**, *upper line*) and case 2 (**b**, *lower line*). US and MRI show a localized mass in each case. Partial mastectomy was performed prior to immediate reconstruction using a free dermal fat graft, which resulted in surgical margins that were negative for cancer in both cases (**a**, **b**, *right panel*)

**Fig. 2:**
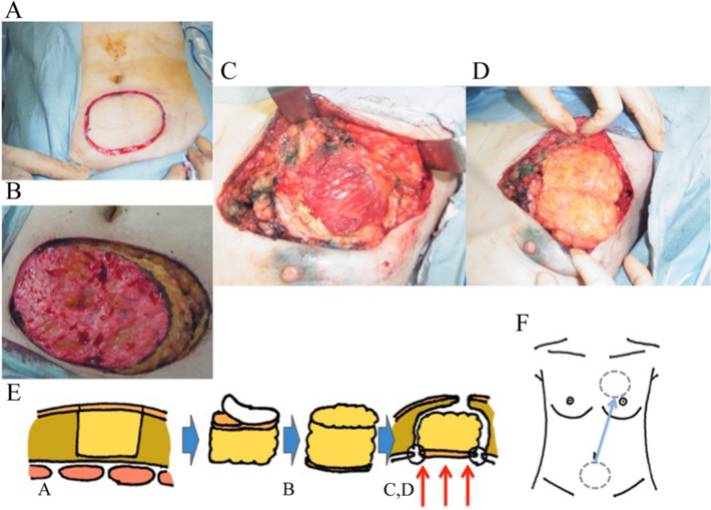
A free dermal fat graft (FDFG: 7.0 cm × 9.0 cm) was harvested from the lower abdomen (**a**). The specimen is denuded of epidermal skin and made into a columnar shape (**b**). The breast defect (**c**) was repaired and filled with the FDFG, with the denuded surface fitted to the pectoralis major muscle (**d**). In the semi-sitting position, nipple and breast symmetry are checked carefully. A schematic diagram of this process is shown (**e**, **f**)

**Fig. 3:**
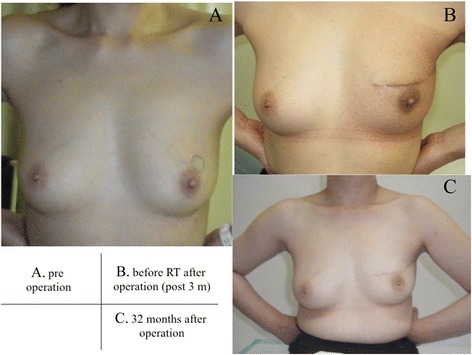
Pictures indicated temporal statuses of Case 1; a status before operation (**a**), post-operative status before radiation therapy (3 months after operation) (**b**), and 32 months after operation (**c**)

A 46-year-old premenopausal woman was diagnosed with T1N0M0 clinical stage I breast cancer. Her body mass index (BMI) was 22.5 kg/m^2^. The tumor was seen as a focal asymmetry density on mammography, a 1.8-cm heterogeneously hypoechoic, microlobulated mass on US, and a localized enhanced mass in the early period on magnetic resonance imaging (MRI) in the upper region of her left breast, as shown in the left and center panels of Fig. 1a. Sentinel lymph node biopsies (SNBs) were performed using ^99m^technetium-phytic acid and indigo carmine solution to find the SNs. No metastatic cancer was detected in the three extracted SNs. Immediate reconstruction of the surgical defect was performed using an autologous FDFG from the lower abdomen (Fig. 2a) as a volume replacement technique [4, 5, 7, 8]. The FDFG (7.0 cm × 9.0 cm) was harvested as a columnar-shaped specimen (Fig. 2b). The FDFG was implanted into the defect with its dermis facing the surface of the pectoralis major muscle turning it upside-down after de-epithelialization (Fig. 2c). The margin of the dermis of the FDFG was then sutured to the edge of the fascia of the pectoralis major muscle using 3-0 Vicryl (Ethicon, Inc, Somerville, NJ; Fig. 2d). This process is shown in Fig. 2e, f. After breast symmetry was balanced, the subcutaneous tissue and skin were sutured using 3-0 Vicryl and 4-0 PDS-II (Ethicon, Inc). Closed suction drains were placed onto the surfaces of the implanted FDFG and sub-dermal space. On pathological examination, the tumor consisted of a 1-mm micro-invasive ductal carcinoma (IDC) with 5 cm of intra-ductal component, and surgical margins were negative (Fig. 1a, right panel); the tumor was positive for estrogen receptor (ER) and progesterone receptor (PgR) and negative for human epidermal growth factor 2 (HER2) and lymphatic vessel invasion, with a nuclear grade (NG) score of 2 and Ki67 of 10 %. Radiation therapy (RT; 50 Gy in 25 fractions with a boost of 12.5 Gy in five fractions to the tumor bed) and tamoxifen were given as adjuvant therapy. The patient has been recurrence-free for 44 months following surgery, and her appearance during the process is shown in Fig. 3a–c.

**Fig. 4:**
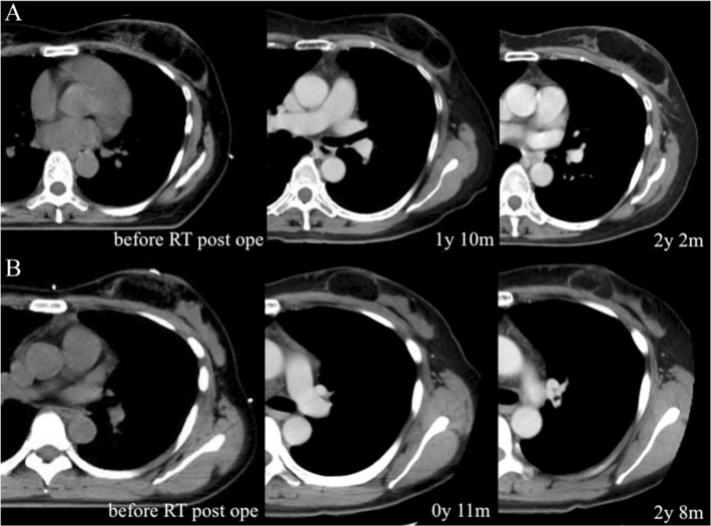
Chronological changes of grafts in case 1 (**a**, *upper line*) and case 2 (**b**, *lower line*). After 2 years and more, shrinkage of grafts was 21.6 and 25.2 %, respectively. Slight enhancement in the center of or around the graft is indistinct in each case, and it is difficult to assess the blood flow in detail

**Fig. 5:**
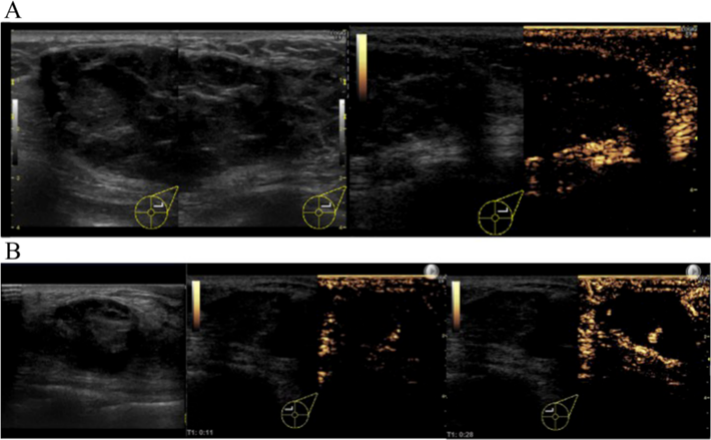
Ultrasound (US) and contrast-enhanced ultrasound (CEUS) were performed in case 1 (**a**, *upper line*) and case 2 (**b**, *lower line*) over 3 years after surgery. In case 1, the graft is detected as a circumscribed oval and heterogeneous low echoic mass on US. This mass is coated by a smooth capsule with a high echogenic structure (**a**, *left panel*). CEUS shows no blood flow into the mass from the outside fat tissue (**a**, *right panel*). In case 2, the graft is seen as a circumscribed oval and heterogeneous low echoic mass on US, very similar to case 1. CEUS shows blood flow into the graft mainly from the retro-mammary fat side, which begins approximately 10 s after intravenous administration of contrast agent (*center panel*). In the mass, several blood flows are observed with persistent enhancement without attenuation once the vessels are enhanced for 30 s after administration (*right panel*)

On the questionnaire, a “good” score (10 of 12 points) was obtained both 6 months and 3 years after the operation; 2 points were lost due to wound scarring and softness of the breast (Table 1). Twenty-six months after the operation, this patient underwent 64-detector-row CT (LightSpeed VCT VISION; GE Healthcare, Milwaukee, WI, USA). The size of the graft was measured using the slice in which the largest FDFG was present on CT. There was little change in FDFG thickness, from 20 to 22 mm, and FDFG width decreased from 68 to 65 mm (dimension decrease rate 21.6 %), 26 months after surgery, as shown in Fig. 4a. The FDFG was detected as a circumscribed oval and heterogeneous low echoic mass on US 40 months after the operation. This mass was coated by a smooth capsule with a high echogenic structure (Fig. 5a, left panel). CEUS showed no blood flow into the mass from the outside fat tissue (Fig. 5a, right panel).

### Case 2

**Fig. 6:**
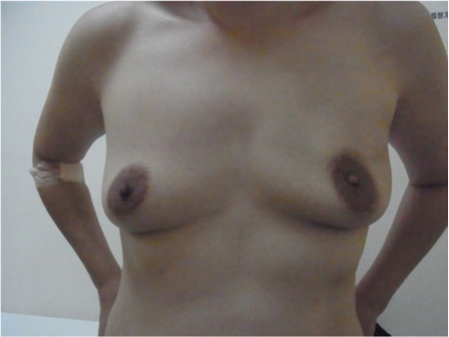
Postoperative appearance of case 2. A 37-year-old woman with a localized 1.2-cm tumor in the upper region of the left breast 44 months after surgery

A 37-year-old premenopausal woman with a localized mass in the inner-upper quadrant of her left breast was diagnosed as having T1N0M0 clinical stage I breast cancer. Her BMI was 20.9 kg/m^2^, less than case 1. The tumor was seen as a circumscribed equal-density mass on mammography, a 1.2-cm, hypoechoic, microlobulated mass on US, which was visualized as an enhanced localized mass in the upper region of her left breast in the early period on MRI, as shown in the left and center panels of Fig. 1b. No metastases were found in two SNs. She underwent surgery with an FDFG (4.2 cm × 6.5 cm) in the same manner as previously described. On pathological examination, the tumor was a 1.4-cm IDC and surgical margins were negative (Fig. 1b, right panel), positive for ER and negative for PgR, HER2, and lymphatic vessel invasion, with an NG score of 1 and Ki67 of 30 %. After surgery, RT (50 Gy in 25 fractions with a boost of 12.5 Gy in five fractions) and tamoxifen were administered. She has been free from recurrence for 45 months following surgery, and her appearance is shown in Fig. 6. On the questionnaire, a “good” score (10 of 12 points) was obtained both 6 months and 3 years after operation. As in case 1, 2 points were lost because of wound scarring and softness of the breast, but she commented that she felt the graft region softening somewhat, slowly over the years. FDFG thickness on CT changed from 22 to 19 mm, and FDFG width decreased from 42 to 37 mm (dimension decrease rate 25.2 %) 32 months after surgery, as shown in Fig. 4b. The FDFG was detected as a circumscribed oval and heterogeneously hypo-echoic mass on US 45 months after operation. This mass was coated by a smooth capsule with an iso- or hyperechoic structure, the findings of which were very similar to those of case 1 (Fig. 5b, left panel). CEUS showed blood flow into the graft mainly from the retro-mammary fat side, which began approximately 10 s after intravenous administration of contrast agent (Fig. 5b, center panel). In the mass, several blood flows were observed with persistent enhancement without attenuation once the vessels were enhanced for 30 s after administration (Fig. 5b, right panel).

From 2011 to 2012, several patients with localized early-stage breast cancer in the upper quadrant of the breast underwent successful BCS with immediate reconstruction using autologous FDFGs in our hospital. The two basic criteria for the FDFG technique were as follows: the patients had a localized malignant neoplasm; and the cancer lesion was restricted to the upper-inner or upper-outer quadrant, with adjoining pectoralis major muscle, as described by Kijima et al. [7]. In our hospital, patients who underwent BCS, even with the FDFG technique, routinely underwent adjuvant RT to prevent local recurrence. However, as in the present two cases, no severe adverse events related to the technique were seen, though radiotherapy might cause the breast tissue to be firmer; the breast tissue becomes more fibrous, hard, and less stretchy due to radiation fibrosis [10].

To assess the benefit of this FDFG technique, the degree of cosmetic satisfaction (using a questionnaire), graft shrinkage (measured by CT), and blood flow (using CEUS) were evaluated to assess the graft 3 years after surgery and postoperative radiotherapy. Two cases that underwent these assessments were described in this report, providing important information given the few previous series.

The questionnaire has been commonly used to assess cosmetic statements by patients, as reported by Sawai’s group and supported by the JBCS [11]. In the present two cases, the patients’ self-reported questionnaire results suggested that this technique might be relatively satisfactory, but the graft appeared to be relatively hard, both subjectively and objectively. It is likely that one reason for the slight decrease in satisfaction might have been graft hardness. Mild resorption of the graft was seen to a similar extent on CT in both cases after 2 years; this is consistent with previous reports of FDFG [7].

Both of the present cases had very similar clinical courses, but there was a slight difference in the patients’ comments about “hardness” and in the CEUS findings. While the graft was continuously hard in case 1, it became slightly softer than immediately after the operation and RT in case 2. On the other hand, CEUS was performed to assess the blood flow in the FDFG at over 3 years after the operation in the present two cases. It is noteworthy that CEUS showed two different patterns: blood flow into the graft mainly from the pectoralis major muscle side was observed with persistent enhancement in case 2, while case 1 showed no enhancement. CEUS can detect blood flow more accurately than CT or MRI, because ultrasound contrast media remain within the vasculature [9]. Thus, CEUS objectively demonstrated vascularization in the graft after approximately 3 years after operation in case 2. This finding is consistent with the hypothesis that engraftment might require revascularization from the pectoralis major muscle to the vascular network in the subcutaneous tissue, because an FDFG is not a pedicle flap [4, 12].

It has been reported that the graft might change to fibrotic tissue and finally shrink due to decreased graft perfusion, and there might be no blood flow in the center, but resumption of blood flow might be seen only in the periphery of the graft [12, 13]. The FDFG may have had a pathologically necrotic background, such as graft resorption and epithelial cyst formation [12], which might result in “hardness.” From the questionnaire results, this technique was relatively acceptable for 3 years, except for this particular point. Despite mild shrinkage of the graft, the volume balance of the breasts might not be lost enough to decrease the patients’ satisfaction. In addition to relatively good cosmetic outcomes in both cases despite RT, case 2 was striking because the graft achieved at least partial blood flow from the pectoralis major muscle, which may be a meaningful clue to investigating such grafts in detail in the near future. However, careful attention is required to use this technique because the abdominal region is well known as a good donor site for free tissue transplantation with blood vessels. This technique appears to be an optional surgical procedure for use in limited cases.

## Conclusions

Graft status was maintained with a good cosmetic outcome for 3 years by BCS with immediate reconstruction using an autologous FDFG, despite mild shrinkage and hardness of the graft. It is notable that blood flow mainly from the pectoralis major muscle into the graft was observed in the subjectively and objectively softer graft case after more than 3 years.

## Consent

The patients provided informed consent for the publication of this report and any accompanying images.
